# Phylogeny and Comparative Analysis of Chinese *Chamaesium* Species Revealed by the Complete Plastid Genome

**DOI:** 10.3390/plants9080965

**Published:** 2020-07-30

**Authors:** Xian-Lin Guo, Hong-Yi Zheng, Megan Price, Song-Dong Zhou, Xing-Jin He

**Affiliations:** 1 Key Laboratory of Bio-Resources and Eco-Environment of Ministry of Education, College of Life Sciences, Sichuan University, Chengdu 610065, China; xlguo@stu.scu.edu.cn (X.-L.G.); hyzheng@stu.scu.edu.cn (H.-Y.Z.); 2 Sichuan Key Laboratory of Conservation Biology on Endangered Wildlife, College of Life Sciences, Sichuan University, Chengdu 610065, China; meganprice@scu.edu.cn

**Keywords:** Apiaceae, *Chamaesium*, phylogeny, plastid genome, comparative analysis

## Abstract

*Chamaesium* H. Wolff (Apiaceae, Apioideae) is a small genus mainly distributed in the Hengduan Mountains and the Himalayas. Ten species of *Chamaesium* have been described and nine species are distributed in China. Recent advances in molecular phylogenetics have revolutionized our understanding of Chinese *Chamaesium* taxonomy and evolution. However, an accurate phylogenetic relationship in *Chamaesium* based on the second-generation sequencing technology remains poorly understood. Here, we newly assembled nine plastid genomes from the nine Chinese *Chamaesium* species and combined these genomes with eight other species from five genera to perform a phylogenic analysis by maximum likelihood (ML) using the complete plastid genome and analyzed genome structure, GC content, species pairwise Ka/Ks ratios and the simple sequence repeat (SSR) component. We found that the nine species’ plastid genomes ranged from 152,703 bp (*C. thalictrifolium*) to 155,712 bp (*C. mallaeanum*), and contained 133 genes, 34 SSR types and 585 SSR loci. We also found 20,953–21,115 codons from 53 coding sequence (CDS) regions, 38.4–38.7% GC content of the total genome and low Ka/Ks (0.27–0.43) ratios of 53 aligned CDS. These results will facilitate our further understanding of the evolution of the genus *Chamaesium*.

## 1. Introduction

**Figure 1:**
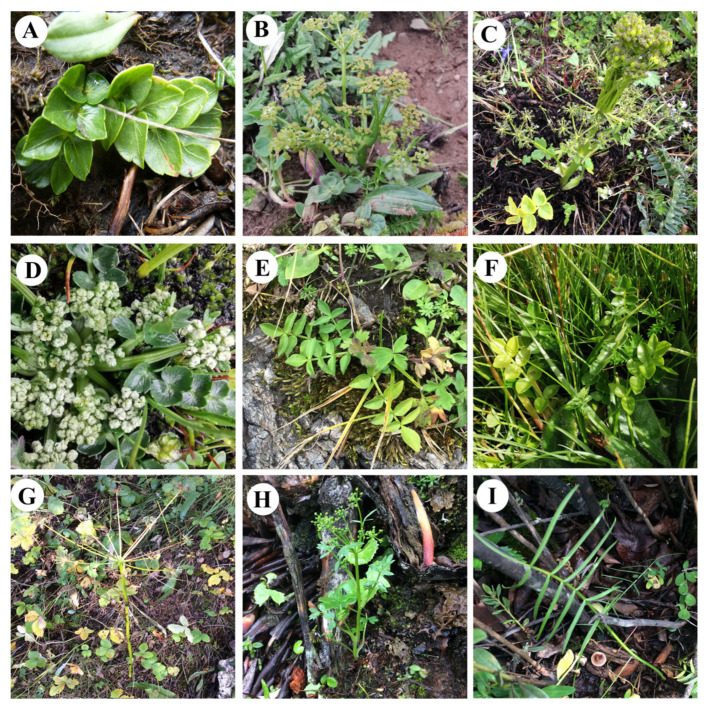
Plants of *Chamaesium*. (**A**) *C. delavayi*, (**B**) *C. jiulongense*, (**C**) *C. mallaeanum*, (**D**) *C. novem-jugum*, (**E**) *C. paradoxum*, (**F**) *C. spatuliferum*, (**G**) *C. thalictrifolium*, (**H**) *C. viridiflorum*, (**I**) *C. wolffianum*.

*Chamaesium* H. Wolff is an endemic genus of Apiaceae, restricted to the Hengduan Mountains and the Himalayas. *Chamaesium* species mostly occur at high altitudes [1,2,3,4,5,6]. Ten species have been identified by distinctive fruits with primary and secondary ribs and 1-pinnate leaf blades (Figure 1). In China, nine species of *Chamaesium* have been described and studied based on morphological characteristics and molecular phylogeny [6,7,8]. *Chamaesium* is monophyletic and occurs at the basal group of Chinese Apiaceae, and we found there were distinct differences between species based on our previous studies [8,9,10]. Although clear inter-specific relationships were described, there are unresolved aspects about the genus, including accurate analysis of the plastid genome, micro-morphology and complex evolutionary issues.

Plastids are significant organelles in plants, and plastid DNA is often more conserved than the nuclear and the mitochondrial genomes [11]. A quadripartite circular structure including two copies of inverted repeat (IR) regions, a large single copy (LSC) region and a small copy region (SCR) usually occur in most angiosperm plastid genomes [12,13]. With the diffusion of next-generation sequencing, whole plastid genome sequences can be assembled with greater ease than with Sanger sequencing. This provides more opportunity for comparative analyses and much greater phylogenetic resolution than traditional gene fragments [14,15,16]. Regardless of the taxonomic level, plastid genome-scale data provide an effective and comprehensive approach to distinguishing species [17,18,19,20,21].

Since there is still uncertainty regarding certain aspects of *Chamaesium*’s phylogeny, our study aimed to investigate the plastid sequences of the nine *Chamaesium* species in China. We aimed to provide (1) eight complete plastid genome sequences, (2) comparative analyses of the nine plastid genome sequences and (3) phylogenetic analyses of nine plastid genome sequences. The complete plastid genome sequences of *Chamaesium* provide effective data to enhance understanding of these plants distributed in the Himalayas and the Hengduan Mountains.

## 2. Results

### 2.1. The Plastid Genome of Chamaesium Species

**Figure 2:**
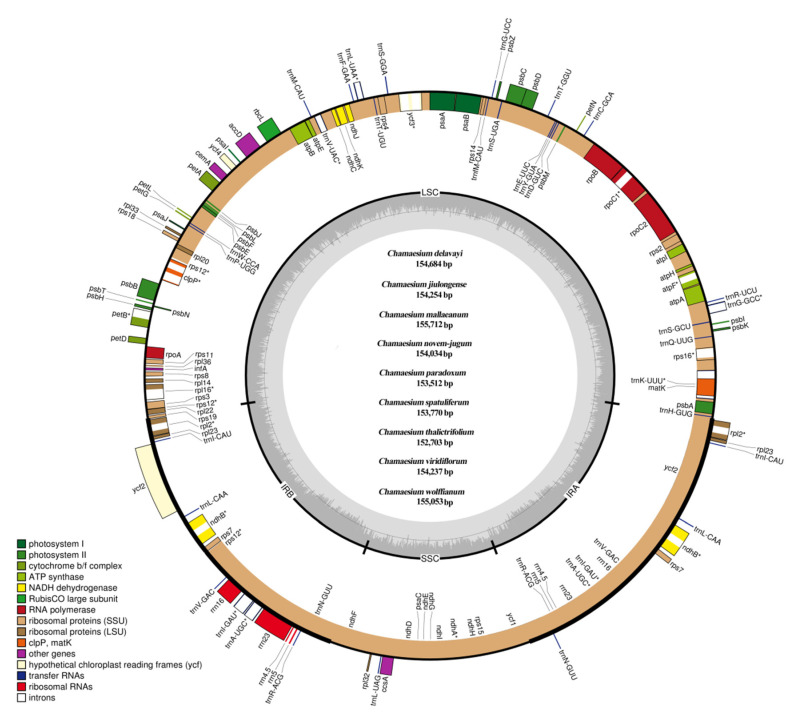
Plastid genome map of *Chamaesium*. The genes shown inside and outside of the circle are transcribed in the clockwise and counterclockwise directions, respectively. Genes belonging to different functional groups are drawn in different colors. The darker grey area in the inner circle indicates the GC content, while the lighter grey corresponds to the AT content. LSC, large single copy; SSC, small single copy; IR, inverted repeat.

The complete plastid genome of *Chamaesium* spp. exhibited a single and typical quadripartite circular structure (Figure 2). The sizes of the nine species’ plastid genomes ranged from 152,703 bp (*C. thalictrifolium*) to 155,712 bp (*C. mallaeanum*). The *Chamaesium* plastid genome contained two identical IRs (IRa and IRb, with lengths 25,727–26,147 bp), which were separated by LSC (84,082–85,998 bp) and small single copy (SSC) (17,167–17,580 bp) regions. Different GC content occurs in the whole plastid genome, LSC, SSC and IR regions of the nine *Chamaesium* species. Higher GC content was detected in the IRs compared to the other regions, which was possibly caused by the presence of rRNA sequences (55.20%–55.30%) with high GC content in IRs (Supplementary Table S2).

The plastid genomes of all species contained 133 genes, including ca. 85 protein-coding genes (PCGs), 37 transfer RNA genes (tRNA) and eight ribosomal RNA genes (rRNA) (Table 1). Among these genes, 95 genes were unique, while 19 genes were duplicated in the IR regions, including eight protein-coding genes (*ndhB*, *rpl2*, *rpl23*, *rps7*, *rps19*, *ycf1*, *ycf2*, *ycf15*), seven tRNA genes (*trnA-UGC*, *trnI-CAU*, *trnI-GAU*, *trnL-CAA*, *trnN-GUU*, *trnR-ACG* and *trnV-GAC*) and four rRNA genes (*rrn4.5*, *rrn5*, *rrn16* and *rrn23*). In addition, 11 genes (*atpF*, *ndhA*, *petB*, *rpl16*, *rpoC1*, *rps12*, *rps16*, *trnG-GCC*, *trnK-UUU*, *trnL-UAA*, *trnV-UAC*) contained one intron, and six genes (*clpP*, *ndhB*, *rpl2*, *trnA-UGC*, *trnI-GAU*, *ycf3*) contained two introns. Four pseudogenes (*ψ rps19*, *ψ ycf1* and two *ψ ycf15)* were found in all nine species.

The PCGs in the *Chamaesium* plastid genome included five genes encoding photosystem I subunits (*psaA*, *psaB*, *psaC*, *psaI*, *psaJ*), while 15 genes were related to photosystem II subunits (*psbA*, *psbB*, *psbC*, *psbD*, *psbE*, *psbF*, *psbH*, *psbI*, *psbJ*, *psbK*, *psbL*, *psbM*, *psbN*, *psbT*, *psbZ*). We found nine genes encoding large ribosomal proteins (*rpl2*, *rpl14*, *rpl16*, *rpl20*, *rpl22*, *rpl23*, *rpl32*, *rpl33*, *rpl36*) and 12 genes encoding small ribosomal proteins (*rps2*, *rps3*, *rps4*, *rps7*, *rps8*, *rps11*, *rps12*, *rps14*, *rps15*, *rps16*, *rps18*, *rps19*). Additionally, we found six genes (*atpA*, *atpB*, *atpE*, *atpF*, *atpH*, *atpI*) that encoded ATP synthase and electron transport chain components.

### 2.2. Contraction and Expansion of IRs and Simple Sequence Repeat (SSR) Analysis

**Figure 3:**
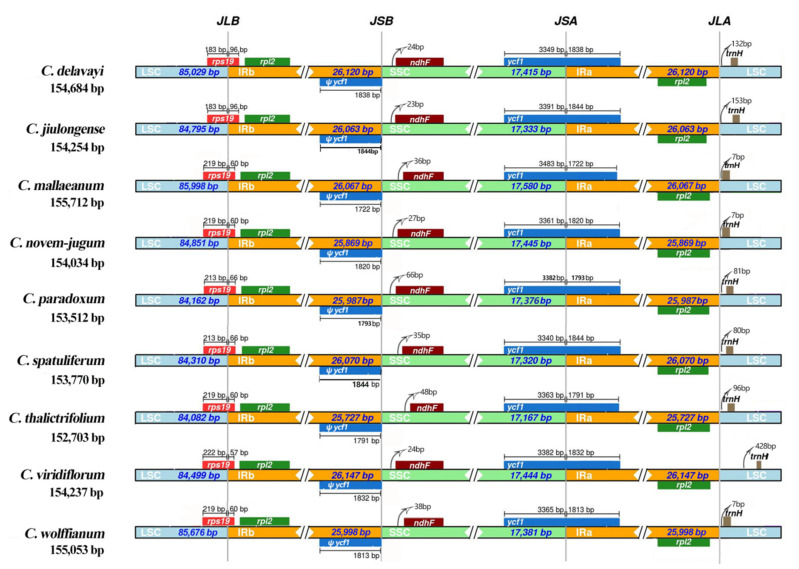
Comparisons of LSC, SSC and IR region borders among nine *Chamaesium* plastid genomes.

The IR boundaries of the nine *Chamaesium* plastid genomes were compared to observe the expansions and contractions in this region (Figure 3). Despite the plastid genome of these nine *Chamaesium* species showing a similar structure, some variations were identified. The IRb region expanded to the *rps19* gene with 96 bp, 96 bp, 60 bp, 60 bp, 66 bp, 66 bp, 60 bp, 57 bp and 60 bp in *Chamaesium delavayi*, *C. jiulongense*, *C. mallaeanum*, *C. novem-jugum*, *C. paradoxum*, *C. spatuliferum*, *C. thalictrifolium*, *C. viridiflorum* and *C. wolffianum,* respectively. The *ndhF* genes of these nine species are encompassed entirely in the SSC region, and a 23–48 bp length of the intergenic region exists between the JSB line (the border between IRb and SSC) and the *ndhF* gene. The *ycf1* gene occupies the SSC and IRa regions (JSA line), with a distance ranging from 1722 to 1844 bp, and is located in the IRa region across the nine species. This also created a corresponding *ycf1* pseudogene at the JSB line. The JLA line was located near the region of the *trnH* gene. The lengths of intergenic space varied between species. *C. viridiflorum* had the longest intergenic space among these species with 428 bp, whereas *C. mallaeanum*, *C. novem-jugum* and *C. wolffianum* had only 80 bp.

We identified 34 SSR types and 585 SSR loci across the nine species, ranging from 63 SSRs (*C. paradoxum*, *C. spatuliferum* and *C. thalictrifolium*) to 80 SSRs (*C. wolffianum*) (Supplementary Table S3). Six compound formations of microsatellites (mono-, di-, tri-, tetra-, penta- and hexanucleotide repeats) were found in the plastid genome of the nine *Chamaesium* species. The most abundant SSRs were mononucleotide repeats, which accounted for 70.94% of all SSRs. Dinucleotide SSRs (15.90%) were the second most common repeat motif, followed by tetranucleotide repeats (8.21%) and trinucleotide repeats (3.59%). Pentanucleotide and hexanucleotide repeats were the least abundant (1.03% and 0.34%).

In *C. mallaeanum* and *C. paradoxum*, mononucleotide repeats were composed entirely of A/T, while other mononucleotide repeated motifs, G/C, were uncommon (0.96%, 1.93%). Most dinucleotide repeats were AT/TA (94.62%), and all dinucleotide repeats found in *C. delavayi*, *C. jiulongense*, *C. spatuliferum* and *C. thalictrifolium* were composed of A/T (Supplementary Table S3).

**Figure 4:**
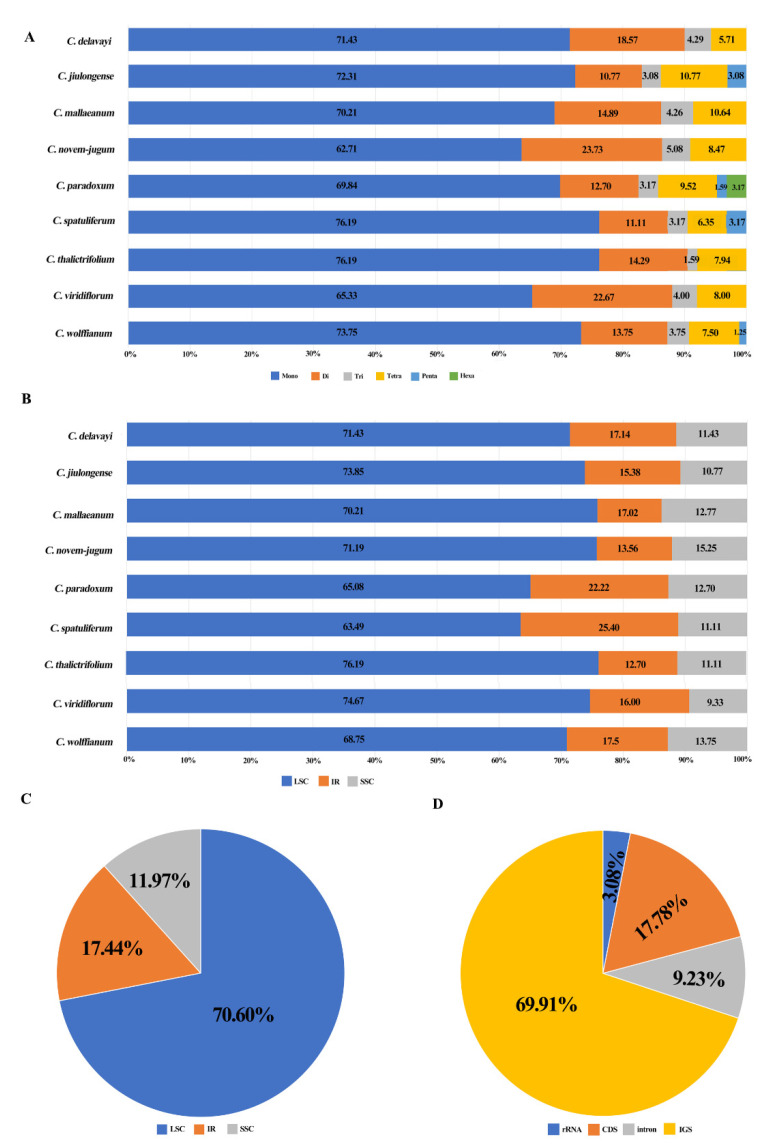
Analysis of simple sequence repeats (SSRs) in nine *Chamaesium* plastid genomes. (**A**) Frequency of identified SSR motifs in different repeat type classes in nine species, (**B**) frequency of identified SSR motifs in LSC, IR, SSC of nine species, (**C**) proportion of repeats in LSC, IR, SSC in total, (**D**) proportion of repeats in tRNA or rRNA, CDS, intron and intergenic spacer (IGS) in total.

Across all SSR loci, 413 SSRs (70.60%), 102 SSRs (17.44%) and 70 SSRs (11.97%) were detected in the LSC, IRs and SSC regions of the plastid genome, respectively. It was found that 176 SSRs were located in 23 gene regions (*atpB*, *atpF*, *cemA*, *clpP*, *ndhA*, *ndhE*, *ndhF*, *ndhJ*, *rpl16*, *rpoA*, *rpoB*, *rpoC1*, *rpoC2*, *rps16*, *ycf1*, *ycf2*, *ycf3*, *trnA-UGC*, *trnG-GCC*, *trnI-GAU*, *trnK-UUU*, *trnL-UAA*, *trnV-UAC*) (Supplementary Table S3). SSRs were also detected in coding sequence (CDS) regions of the *Chamaesium* plastid genome. The CDS regions accounted for 49.80%–51.6% of the overall length. Approximately 69.91% of SSRs were found in non-coding regions, whereas only 17.78% of SSRs were in *Chamaesium* CDS regions (Figure 4).

### 2.3. Codon Usage Bias of Chamaesium Species and Ka/Ks Ratios of Species Pairwise

**Figure 5:**
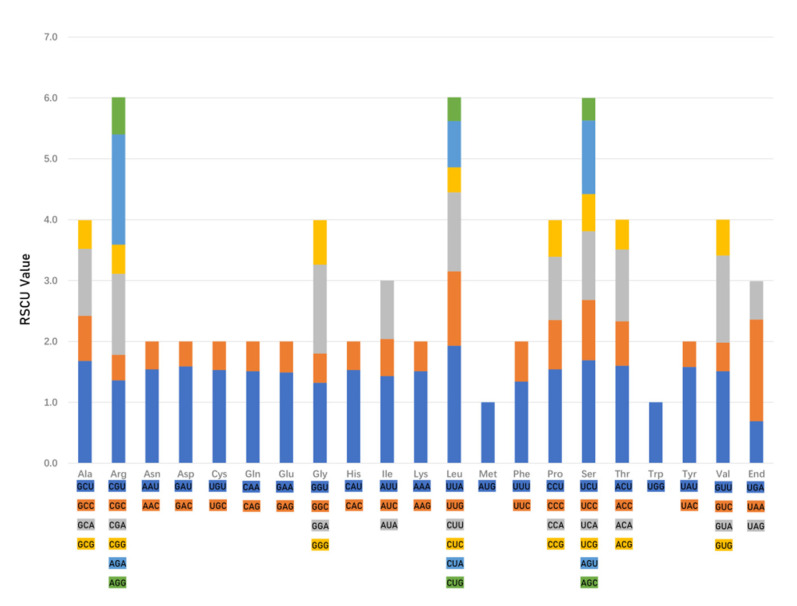
Codon content of 20 amino acid and stop codon in 53 coding genes of the *Chamaesium* plastid genome. The color of the histogram corresponds to the color of codons.

The codon usage frequency and relative synonymous codon usage (RSCU) were analyzed based on 53 protein-coding sequences in the nine *Chamaesium* species’ plastid genomes (Figure 5 and Supplementary Table S4). The frequency of codon usage in these nine species was similar. The number of codons in protein-coding regions ranged from 20,953 (*C. thalictrifolium*) to 21,115 (*C. mallaeanum*). Among these codons, leucine, encoded by 2204–2235, and cysteine, encoded by 218–225, occupied the maximum and minimum of coded amino acids. AUU (838–854) encoding isoleucine and UAG (11–13) encoding a termination codon were the most and least used codons. Codon usage was biased towards A and T at the third codon position in these nine species. Furthermore, 31 codons were detected with an RSCU value of more than 1, indicating that they were the preference codons in the *Chamaesium* plastid genome. Among these 31 codons, only UGG, AUG and UUG ended with guanine, whereas other codons terminated in A/T, and no cytosine was found in the third position.

**Figure 6:**
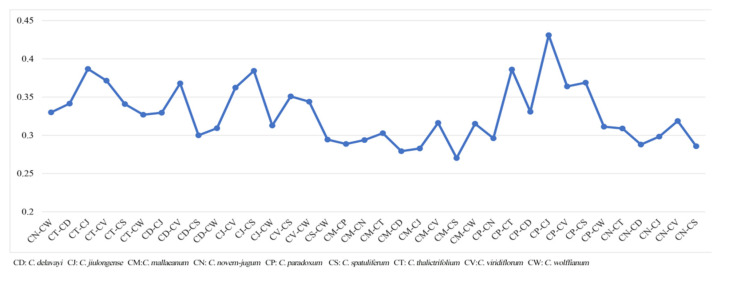
Pairwise Ka/Ks ratios in Chamaesium (Apiaceae). **CD:** *C. delavayi*, **CJ:** *C. jiulongense*, **CM:** *C. mallaeanum*, **CN:** *C. novem-jugum*, **CP:** *C. paradoxum*, **CS:** *C. spatuliferum*, **CT:** *C. thalictrifolium*, **CV:** *C. viridiflorum*, **CW:** *C. wolffianum*.

The Ka/Ks ratios of the nine species (Supplementary Table S5) provided key information on selective pressure that had taken effect in protein-coding sequences. We found pairwise Ka/Ks ratios ranging from 0.27 to 0.43 in comparisons of the nine *Chamaesium* (Apiaceae) species (Figure 6). The highest ratio (0.43) was found when comparing *C. paradoxum* and *C. jiulongense*, while the lowest ratio (0.27) occurred between *C. mallaeanum* and *C. spatuliferum*. All the ratios involved were below 0.5. Therefore, the conservation of plastid protein-coding sequences was confirmed in the genus *Chamaesium*.

### 2.4. Phylogeny of Chamaesium

**Figure 7:**
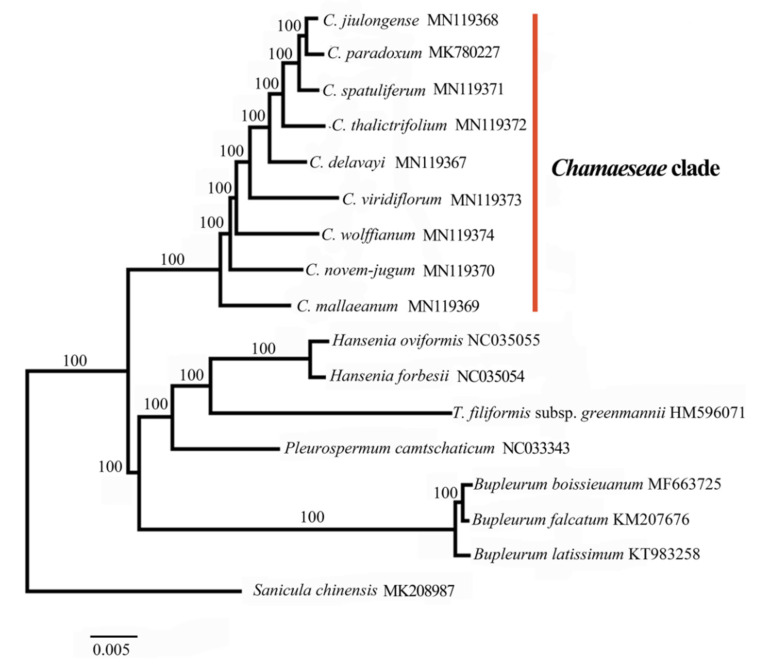
Phylogenetic relationships of *Chamaesium* species with related species based on the whole plastid genomes by maximum likelihood (ML) with bootstrap values above the branches.

Our phylogenetic analysis confirmed that the nine species of *Chamaesium* formed a monophyletic clade (100%) within the *Chamaeseae* clade (Figure 7), which was consistent with the previous study [9,10]. The inter-specific relationships within *Chamaesium* were strongly supported by our analyses. *Chamaesium mallaeanum* was the earliest to speciate, followed by *C. novem-jugum*, *C. wolffianum*, *C. viridiflorum* and *C. delavayi* in order of separation. The latest differentiated taxa, *C. thalictrifolium*, *C. spatuliferum*, *C. paradoxum* and *C. jiulongense*, were very closely related. *C. spatuliferum* is sister to the clade comprising *C. paradoxum* and *C. jiulongense* with a strong support (100%). The topological structure of the maximum likelihood (ML) tree in this study was consistent with previous trees created using gene fragments by maximum parsimony and Bayesian inference [8].

## 3. Discussion

### 3.1. The Fluctuations of IR Regions, Genes and Pseudogenes (ψs) in the Plastid Genome

This study indicates that the lengths of the simple IR region in the nine *Chamaesium* species are very similar (25,727–26,147 bp). The IR region stabilizes and enhances the conserved form of the plastid genome. The high gene conversion ability exists in the plastid genome, ensuring the consistency and stability of the two IR regions [22,23,24]. Generally, IR boundaries between different species are diverse [25]. Fluctuations (expansions and contractions) of the IR regions are the main reasons for the differences in length of the plastid genome, which also causes several genes to enter the IR region or the single-copy sequence [26]. The IR regions of plants such as *Pelargonium hortorum*, *Pisum sativum*, *Cryptomeria fortunei* and *Erodium* spp. are notably inconsistent in length [27,28,29]. In the present study, there was little variation in the length of the two IR regions of the nine *Chamaesium* species, and the simple IR length of ca. 26,000 bp is typical in Apiaceae and other families [30,31,32,33,34].

The total number of *Chamaesium* plastid genes is 133, including pseudogenes. Pseudogenes (ψs) are disabled copies of PCGs and are often referred to as genomic fossils [35,36]. Protein-coding genes will become ψs if degenerated features are present, such as frameshifts, in-frame stop codons and truncations of full-length genes [37,38]. The pseudogene *ψycf1* with ca. 1800 bp length was detected in all *Chamaesium* species in the JSB line, and it has been controversial whether *ycf15*, as a member of this family, has encoding protein properties [39,40,41]. We found that *ycf15* was annotated as a pseudogene in *Foeniculum vulgare* and *Daucus carota* based on previous studies [42] for the existence of many terminators, and this similar structure was also detected in the nine species of *Chamaesium*. The pseudogenes may act on these species’ evolution. More comprehensive evidence is needed to support this hypothesis.

### 3.2. Codon Usage Analysis, Ka/Ks and Selection Pressure

A similar codon (AT) usage bias was found in all nine *Chamaesium* species. A higher AT content at the third codon position was detected in the nine *Chamaesium* species, which was also observed in other terrestrial plant plastid genomes [43,44,45,46]. Furthermore, codons ending with A/T were more common among codons with high RSCU values. These findings are consistent with other reported angiosperm genomes [47,48]. Codon usage biases are related to the carriage of genetic information and proteins with biochemical functions [48,49]. These results on codon usage bias may assist us in better understanding the molecular evolution mechanisms and gene expression in *Chamaesium* species [50,51,52,53].

We found a lack of a sufficient variety of sites at the simple gene level; thus, we aligned 53 CDSs of the nine *Chamaesium* species, and pairwise comparisons found that all ratios were below 0.5. The ratio of Ka/Ks refers to the ratio of the number of non-synonymous substitutions per non-synonymous site (Ka) to the number of synonymous substitutions per synonymous site (Ks) [54]. The ratio can be used as an indicator to measure the selection pressure of the protein-encoding gene [55]. This is equal to the neutral selection; that is, the ratio of observed non-synonymous mutations to synonymous mutations matches the ratio of the expected random mutation model. Therefore, amino acid changes are neither selected nor eliminated. A value greater than 1 indicates that amino acid changes are evolutionarily preferred, meaning that these mutations are more adapted [56,57]. This unusual state may reflect changes in gene function or changes in environmental conditions that force the body to adapt. Consequently, the conservative plastid protein-coding sequences were confirmed in *Chamaesium*, and little variety occurred among these species, especially in key genes.

### 3.3. Phylogenetic Analysis

Our results indicate that the nine species of *Chamaesium* form a monophyletic group (1/100%) in the *Chamaeseae* clade by maximum likelihood (ML) analysis. The inter-specific relationships within *Chamaesium* are strongly supported by our findings. We found that *C. mallaeanum*, which is distributed in the Himalayas, was the earliest differentiated taxon, and the Himalayas is close to the ancestral location of the ancestor *Chamaesium* species. *Chamaesium novem-jugum*, *C. wolffianum*, *C. viridiflorum* and *C. delavayi* are distributed narrowly in Tibet and North Yunnan, radiating out from the Himalayas in the next speciation event. The latest differentiated taxa, *C. thalictrifolium*, *C. spatuliferum*, *C. paradoxum* and *C. jiulongense*, are distributed widely in south Gansu, south Qinghai and west Sichuan. Originating from the Himalayas and spreading to the Hengduan Mountains is the most reliable explanation for the origin of this genus.

## 4. Materials and Methods

### 4.1. Plant Material and DNA Extraction

The nine species investigated in this study were *Chamaesium delavayi*, *C. jiulongense*, *C. mallaeanum*, *C. novem-jugum*, *C. paradoxum*, *C. spatuliferum*, *C. thalictrifolium*, *C. viridiflorum* and *C. wolffianum*. Voucher specimens of these species were deposited in the herbarium of Sichuan University (SZ) (Supplementary Table S1). Fresh leaves were collected from the wild, and they were desiccated and stored in silica gel. Total genomic DNA was extracted from leaf materials, using the modified CTAB procedure [58].

### 4.2. Illumina Sequencing, Assembly, and Annotation

Total genomic DNA was sequenced using an Illumina Novaseq 6000 platform (Illumina, San Diego, CA, USA) with Novaseq 150 sequencing strategy by Novogene (Beijing, China). The remaining clean data were assembled using NOVOPlasty 2.7.1 [59] with K-mer 39, where rbcL of *Chuanminshen violaceum* (GenBank accession No.: KU921430) was used as seed input and the reference sequence. The assembled nine whole plastid genomes were mapped against the reference plastid genome of *Chuanminshen violaceum* using GENEIOUS R11 [60].

### 4.3. Genome Annotation and Repeat Structure

Gene and IR regions annotation of the assembled genomes was undertaken using PGA [61]. Manual modifications for the uncertain start and stop codons were conducted based on comparison with homologous genes from other species’ plastid genomes using GENEIOUS R11. Circular gene maps for these annotated genomes were drawn using the online program of OGDRAW [62]. The nine *Chamaesium* species’ annotated genome sequences were submitted to GenBank, and their corresponding accession numbers are listed in Supplementary Table S1.

### 4.4. Contraction and Expansion of IRs, GC Content and SSR

The boundaries between the IR and SC regions of the nine *Chamaesium* species were compared using the program IRscope. (https://irscope.shinyapps.io/irapp/) [63]. The total GC content and GC content of each region (IR, LSC, SSC) were compared between the nine species based on the program GENEIOUS R11. The plastid SSRs were identified using Perl script MISA [64] with the following repeat threshold settings: 10 repeats for mononucleotide, 5 for dinucleotide, 4 for trinucleotide and 3 repeats for tetra-, penta- and hexanucleotide SSRs.

### 4.5. Phylogenetic Analyses

To infer phylogenetic relationships within *Chamaesium*, the nine plastid genomes were compared to *Bupleurum boissieuanum*, *Bupleurum falcatum*, *Bupleurum latissimum*, *Hansenia forbesii*, *Hansenia oviformis*, *Pleurospermum camtschaticum*, *Tiedemannia filiformis* subsp. *greenmannii* and *Sancula chinense* as outgroups. All plastid genome sequences were aligned using MAFFT v7.402 [65]. Maximum likelihood analyses were conducted using RAxML v7.2.8 [66] with GTR+G, the best-fit model selected by ModelFinder and 1000 bootstrap replicates.

### 4.6. Codon Usage and Ka/Ks Ratios of Species Pairwise Analysis

A total of 53 coding sequences (CDSs) (>300 bp) in the plastid genome of *Chamaesium* were used to analyze the ratios of the synonymous site (Ks) and non-synonymous site (Ka). These 53 CDSs were aligned with MAFFT v. 7 [65]. Manual alterations were performed by MEGA6 [67]. Pairwise Ka/Ks ratios of the nine species were calculated based on the 53 CDS alignments in KaKs Calculator 2.0 [68].

## Figures and Tables

**Table 1:** List of genes encoded in nine *Chamaesium* species.

Group of Genes	Name of Genes
Self-replication	
transfer RNAs	trnA-UGC *, *trnC-GCA*, *trnD-GUC*, *trnE-UUC*, *trnF-GAA*, *trnfM-CAU*,
	*trnG-GCC*, *trnG-UCC*, *trnH-GUG*, trnI-CAU *, trnI-GAU *, *trnK-UUU,*
	trnL-CAA *, *trnL-UAA*, *trnL-UAG*, *trnM-CAU*, trnN-GUU *, *trnP-UGG,*
	*trnQ-UUG*, trnR-ACG *, *trnR-UCU*, *trnS-GCU*, *trnS-GGA*, *trnS-UGA*,
	*trnT-GGU*, *trnT-UGU*, trnV-GAC *, *trnV-UAC*, *trnW-CCA*, *trnY-GUA*
ribosomal RNAs	rrn4.5 *, rrna5 *, rrn16 *, rrn23 *
RNA polymerase	*rpoA*, *rpoB*, *rpoC1*, *rpoC2*
Small subunit of ribosomal proteins (SSU)	*rps2*, *rps3*, *rps4*, rps7 *, *rps8*, *rps11*, *rps12*, *rps14*, *rps15*, *rps16*, *rps18*, rps19 * (*rps19*, *ψrps19*)
Large subunit of ribosomal proteins (LSU)	rpl2 *, *rpl14*, *rpl16*, *rpl20*, *rpl22*, rpl23 *, *rpl32*, *rpl33*, *rpl36*
Genes for photosynthesis	
Subunits of NADH-dehydrogenase	*ndhA*, ndhB *, *ndhC*, *ndhD*, *ndhE*, *ndhF*, *ndhG*, *ndhH*, *ndhI*, *ndhJ*, *ndhK*
Subunits of photosystem I	*psaA*, *psaB*, *psaC*, *psaI*, *psaJ*
Subunits of photosystem II	*psbA*, *psbB*, *psbC*, *psbD*, *psbE*, *psbF*, *psbH*, *psbI*, *psbJ*, *psbK*, *psbL*, *psbM*, *psbN*, *psbT*, *psbZ*
Subunits of cytochrome b/f complex	*petA*, *petB*, *petD*, *petG*, *petL*, *petN*
Subunits of ATP synthase	*atpA*, *atpB*, *atpE*, *atpF*, *atpH*, *atpI*
Large subunit of rubisco	*rbcL*
Other genes	
Translational initiation factor	*infA*
Protease	*clpP*
Maturase	*matK*
Subunit of Acetyl-CoA-carboxylase	*accD*
Envelope membrane protein	*cemA*
C-type cytochrome synthesis gene	*ccsA*
Genes of unknown function	
Hypothetical chloroplast reading frames (ycf)	*ycf1* * (*ycf1,ψycf1*), *ycf2* *, *ycf3*, *ycf4*, ψycf15 *
Total	133

* Duplicated genes, *ψ* shows pseudogenes.

## Supplementary Materials

The following are available online at https://www.mdpi.com/2223-7747/9/8/965/s1, Table S1: Voucher details and GenBank accession numbers of taxa used in this study. Table S2: Summary of complete plastid genome features. Table S3: Statistics of simple sequence repeats in *Chamaesium*. Table S4: Statistics of codon usage bias in *Chamaesium*. Table S5: Comparative analysis of Ka/Ks value in *Chamaesium*.
